# Code sharing in ecology and evolution increases citation rates but remains uncommon

**DOI:** 10.1002/ece3.70030

**Published:** 2024-08-27

**Authors:** Brian Maitner, Paul Efren Santos Andrade, Luna Lei, Jamie Kass, Hannah L. Owens, George C. G. Barbosa, Brad Boyle, Matiss Castorena, Brian J. Enquist, Xiao Feng, Daniel S. Park, Andrea Paz, Gonzalo Pinilla‐Buitrago, Cory Merow, Adam Wilson

**Affiliations:** ^1^ Department of Integrative Biology University of South Florida St. Petersburg Florida USA; ^2^ Department of Geography University at Buffalo Buffalo New York USA; ^3^ Departamento de Ecologia Universidad Nacional San Antonio Abad del Cusco Cusco Peru; ^4^ University at Buffalo Buffalo New York USA; ^5^ Macroecology Laboratory, Graduate School of Life Sciences Tohoku University Sendai Miyagi Japan; ^6^ Center for Global Mountain Biodiversity, Globe Institute University of Copenhagen Copenhagen Denmark; ^7^ Center for Macroecology, Evolution, and Climate, Globe Institute University of Copenhagen Copenhagen Denmark; ^8^ Florida Museum of Natural History University of Florida Gainesville Florida USA; ^9^ Department of Computer Science University of Arizona Tucson Arizona USA; ^10^ Department of Ecology and Evolutionary Biology University of Arizona Tucson Arizona USA; ^11^ The Santa Fe Institute Santa Fe New Mexico USA; ^12^ Department of Biology University of North Carolina at Chapel Hill Chapel Hill North Carolina USA; ^13^ Department of Biological Sciences Purdue University West Lafayette Indiana USA; ^14^ Purdue Center for Plant Biology Purdue University West Lafayette Indiana USA; ^15^ Department of Environmental Systems Science, Institute of Integrative Biology ETH Zürich Zürich Switzerland; ^16^ Département de Sciences Biologiques Université de Montréal Montreal Québec Canada; ^17^ PhD Program in Biology, The Graduate Center and Department of Biology, City College of New York City University of New York New York New York USA; ^18^ Eversource Energy Center and Department of Ecology and Evolutionary Biology University of Connecticut Storrs Connecticut USA

**Keywords:** code sharing, open access, open data, open science, R software, reproducibility

## Abstract

Biologists increasingly rely on computer code to collect and analyze their data, reinforcing the importance of published code for transparency, reproducibility, training, and a basis for further work. Here, we conduct a literature review estimating temporal trends in code sharing in ecology and evolution publications since 2010, and test for an influence of code sharing on citation rate. We find that code is rarely published (only 6% of papers), with little improvement over time. We also found there may be incentives to publish code: Publications that share code have tended to be low‐impact initially, but accumulate citations faster, compensating for this deficit. Studies that additionally meet other Open Science criteria, open‐access publication, or data sharing, have still higher citation rates, with publications meeting all three criteria (code sharing, data sharing, and open access publication) tending to have the most citations and highest rate of citation accumulation.

## Graphical abstract

Biologists increasingly rely on computer code to collect and analyze their data, reinforcing the importance of published code for transparency, reproducibility, training, and a basis for further work. Here, we conduct a literature review examining temporal trends in code sharing in ecology and evolution publications since 2010, and test for an influence of code sharing on citation rate. We find that scientists overwhelmingly (94%) do not publish their code with little improvement over time. We also found there may be an incentive to share, as we additionally demonstrate code sharing may improve citation rate, particularly when combined with open‐access publication and data sharing.

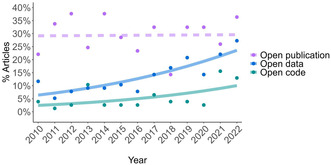

## 1 INTRODUCTION

Reproducibility and transparency are cornerstones of reputable, rigorous, and mature science (Freedman et al., 2015; McNutt, 2014; Munafò et al., 2017; Nosek et al., 2015; “Reality check on reproducibility”, 2016). While definitions can vary by disciplines or change over time (Baker, 2016), reproducibility commonly refers to the ability to repeat previous analyses and obtain consistent results. Transparency refers to the degree or extent that details of the work, especially the methods and results, are openly documented and communicated, thus allowing alternative interpretations (Drescher & Edwards, 2019; Wagenmakers et al., 2021). For programming scripts, the reproducibility spectrum begins with public, permanently archived code (Parker et al., 2016; Peng, 2011). In ecology and evolution, code (primarily in the statistical programming language R; Lai et al., 2019; R Core Team, 2023) has become the basis of most analyses (Feng et al., 2020), and the benefits of code sharing are increasingly recognized (Munafò et al., 2017; Parker, Forstmeier, et al., 2016). Clear, reusable code released under a permissive license (Stodden, 2009) ensures better reproducibility of analyses, but it also may enhance the impact of publications (e.g., greater uptake of methods, more citations) and reduce duplicated efforts, allowing science to progress more effectively (McNutt, 2014; Munafò et al., 2017; Nosek et al., 2015; “Reality check on reproducibility”, 2016). Furthermore, well‐documented code facilitates the peer review process, provides a valuable educational resource (Busjahn & Schulte, 2013), and facilitates our ability to credit developers, as software and package‐usage data can be harvested directly from published code (Merow, Boyle, et al., 2023).

Has the increasing appreciation of code sharing influenced code sharing practices over time? Recent evidence suggests that biologists may be reluctant to share code. A study focused on publications in ecology journals with policies that mandated or encouraged code sharing found that 73% failed to share code (Culina et al., 2020), while a study focused on publications using agent‐based models found that 81% did not provide code (Barton et al., 2022). PLOS open science indicators likewise suggest that code sharing is rare, with 92% of publications in Agricultural and Biological Sciences failing to share code (in comparison, 49% fail to share data; Public Library of Science, 2023). While some papers include the statement “code available upon request,” this promise is often not met (Stodden et al., 2018). Where published, code may also not be reusable due to licensing issues (Stodden, 2009). Resistance to code sharing and reuse may arise from unfamiliarity with best sharing practices, insecurity about code quality, fears of misuse or unsolicited appropriation of ideas, and excess preparation costs (Cadwallader & Hrynaszkiewicz, 2022; Gomes et al., 2022). However, it has been argued that many perceived issues with code sharing stem from misunderstandings of its risks and benefits (Gomes et al., 2022). To better understand how code sharing practices have changed over time and whether code sharing actually improves citation rates, we (1) estimated trends in R code sharing for articles in ecology and evolution published between 2010 and 2022 and (2) tested whether the citation rate was higher for papers that shared code. We focus on R because it has become the dominant coding language in ecology and evolution (Lai et al., 2019).

## 2 MATERIALS AND METHODS

### 2.1 Data collection

#### 2.1.1 List of ecology and evolution publications citing R

To generate a list of papers in ecology and evolution that likely made use of the R programming language (R Core Team, 2023), we performed a query on the Scopus database (https://www.scopus.com) using the *rscopus* R package (Muschelli, 2019). We searched Scopus (performed August 19, 2022) for peer reviewed journal articles that: (1) included the words “ecology” or “evolution” in an “all fields” search (which searches article titles, keywords, abstracts, and journal titles); (2) were published in journals within the subject area “agriculture and biological sciences”; (3) were published after January 1, 2010; (4) were written in English (as this is currently the dominant language of publication in ecology and evolution; Mauranen et al., 2010); and (5) included a citation of R in their reference list.

#### 2.1.2 Checking for code and data availability

We manually evaluated a randomly chosen subset of the publications on our overall list. We selected a total of 1001 papers, evenly distributed across the time period (77 per year * 13 years). Papers that cited R but did not use it (or were unclear on whether they used it; *n* = 3) were discarded and replaced by a randomly selected paper from the same year. For each publication in this subset, we manually identified whether the publication shared any R code, either as supplementary information, or via a link (e.g., to a Github repository). For each paper, we (i) checked for the presence of code in supplemental material, (ii) skimmed publications for code and data availability statements, (iii) searched through publications for terms associated with code (i.e., “code”, “supplement”, “appendix”, “R”, “script” “Github”), and (iv) searched publications for URLs. Papers were scored with a binary variable indicating whether they shared R code or not. We did not distinguish between publications which shared sufficient code for reproduction and those which did not. We also did not attempt to rerun the code or assess its reproducibility, and only recorded the presence of any code, even if it was incomplete. Where code was included, we recorded the license the code was provider under, or lack thereof. We also assessed whether publications were open access and whether they shared open data in order to understand the importance of open code relative to these other open‐access components. Open access information was provided by the *rscopus* R package (Muschelli, 2019). Open data was scored as a binary variable indicating whether the authors shared the full set of raw data underlying the analyses or not. To control for differences in citation rates among journals, we downloaded impact factor information using the *scholar* R package (Keirstead, 2016) on June 16, 2023. To estimate the proportion of publications which use R but do not properly cite it, we screened 130 randomly selected publications evenly distributed across the time period. These publications were selected using identical criteria to the publications that did cite R, except that they did not include R in their list of references.

### 2.2 Checking for code citations

Where code was shared in a citable location such as a DOI or URL (*n* = 33), we assessed whether the code itself was cited by querying the Scopus database for the URL (and DOI, where appropriate) using the *rscopus* R package (Muschelli, 2019). Publications where code was shared in appendices or supplementary information (*n* = 22) were excluded, as there was no way of distinguishing citations of the code with citations of the publication itself.

### 2.3 Analyses

All analyses were conducted in R version 4.3.0 (R Core Team, 2023). All R scripts underlying these analyses are available at: https://github.com/bmaitner/R_citations and via Zenodo (Maitner & Lei, 2024). For data processing, we used the R packages *stringdist* version 0.9.12 (van der Loo, 2014), *tidyverse* version 2.0.0 (Wickham et al., 2019), *googledrive* version 2.1.1 (D'Agostino McGowan & Bryan, 2023), and *googlesheets4* version 1.1.1 (Bryan, 2023); for analyses, the packages *bbmle* version 1.0.25.1 (Bolker & R Development Core Team, 2022), *DHARMa* version 0.4.6 (Hartig, 2022), *MuMIn* version 1.47.5 (Barton, 2023), *rscopus* version 0.6.6 (Muschelli, 2019), *rsq* version 2.6 (Zhang, 2018), *scholar* version 0.2.4 (Keirstead, 2016), and *stats* version 4.3.0 (R Core Team, 2023); for plotting, the packages *ggplot2* version 3.4.4 (Wickham, 2016), *ggpmisc* version 0.5.5 (Aphalo, 2022), and *questionr* version 0.7.8 to identify all R packages used.

#### 2.3.1 Proportion of papers sharing code over time

We tested for a trend in code‐sharing over time by modeling code sharing (binary, yes/no) as a function of the year (relative to 2010) using a generalized linear model. Modeling was performed using the function *glm* in the *stats* R package (R Core Team, 2023) with a binomial error distribution. We similarly tested for temporal trends in two other open‐science components, open‐access publication (binary) and open data (binary). We also tested whether open‐access or open‐data papers were disproportionately likely to share code using chi‐square tests via the *chisq.test* function in the *stats* R package (R Core Team, 2023).

#### 2.3.2 Impact of code sharing on citations

We additionally modeled the relationship between code sharing and citation count using generalized linear models in R. We modeled the dependent variable (cumulative number of citations of each article by 2022) using a Poisson distribution, which models the number of independent events occurring within a period of time (Bolker, 2008). In addition to the predictor variable for code sharing (binary, yes/no), we included other variables that were hypothesized to influence citation count. Data sharing (binary, yes/no) may increase citation counts as readers may cite papers as data sources (Christensen et al., 2019; Piwowar et al., 2007). Open access (binary, yes/no) may also increase citation counts by reaching a broader set of readers (Tang et al., 2017). Publications accumulate citations over time, and so citation count should increase with publication age (continuous, 1–13 years). Finally, publications in higher impact journals may be more likely to be read and cited, and hence, journal impact factor (continuous, 0–11.633) may be positively associated with citation count. In addition to main effects, we considered two classes of interactions: (1) interactions between publication age and other main effects, which are appropriate if a main effect modifies the rate at which a publication accumulates citations over time; and (2) interactions between open‐science criteria (i.e., open access, open code, and open data), which are appropriate if there are synergistic effects of meeting multiple open‐access criteria. We compared 11 models (including one null model) that differed in complexity and that represented different hypotheses regarding the factors that influence citations (Table 1). Continuous variables were scaled and centered. Overall model pseudo‐*R* ^2^ for the best‐performing model was calculated using the function *r.squaredGLMM* in the *rsq* package (Zhang, 2018).

**TABLE 1:** Candidate models of citation count.

ID	Models	df	ΔAIC
1	Citations ~ Impact factor × Age + R code shared × Age + Open access × Age + Data shared × Age + Data shared × R code shared + R code shared × Open Access + Open access × Data shared	13	0.0
2	Citations ~ Impact factor × Age + R code shared × Age + Data shared × Age + Data shared × R code shared	9	834.0
3	Citations ~ Impact factor × Age + R code shared × Age + Open access × Age + Open access × R code shared	9	907.5
4	Citations ~ Impact factor × Age + R code shared × Age + Open access × R code shared	8	913.0
5	Citations ~ Impact factor × Age + R code shared × Age + Open access × Age + Data shared × Age	10	937.8
6	Citations ~ R code shared + Data shared + Open access + Age + Impact factor	6	1116.9
7	Citations ~ Impact factor × Age + R code shared × Age + Open access × Age	8	1181.5
8	Citations ~ Impact factor × Age + R code shared × Age	6	1339.7
9	Citations ~ Impact factor × Age	4	1973.8
10	Citations ~ Age	2	5824.3
11	Citations ~1	1	13721.6

## 3 RESULTS

**FIGURE 1:**
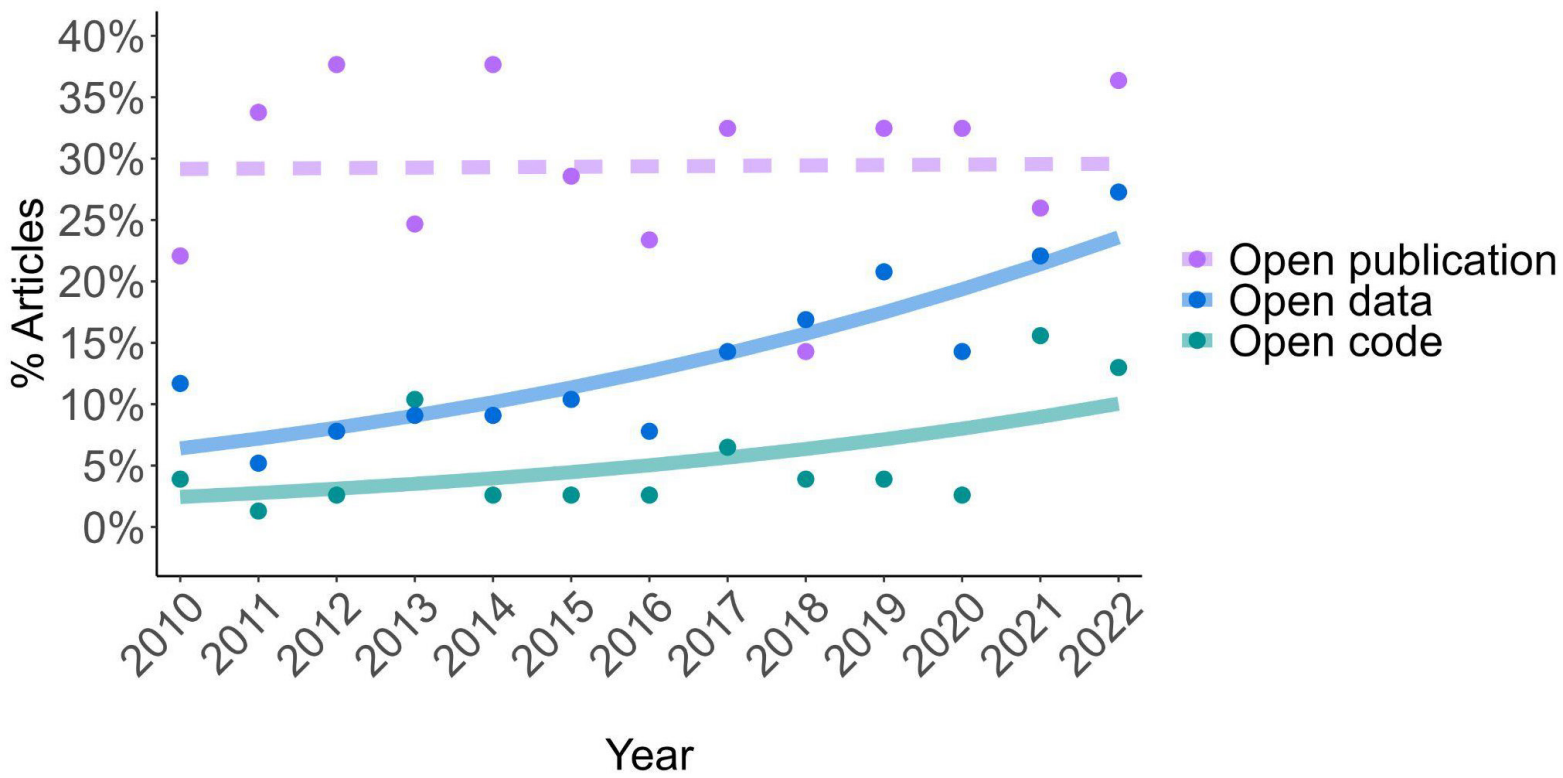
Temporal trends of open‐access publications (open publication), open data, and code sharing (open code) between 2010 and 2022. Sample size was 77 papers per year, 1001 total. The lines show trends in each open‐science component over time, with dashed lines representing non‐significant trends, and solid lines indicating significance. Open data (*p* = 1.48e‐06) and code sharing (*p* = .00157) increased significantly over time, while open‐access publications (*p* = .926) had no statistically significant relationship. Note that publications may be converted to open access after publication as journal policies change, so the open‐publication trend should be interpreted with caution.

We identified 28,227 articles that met our search criteria. From this set of articles, we randomly selected 1001 papers (the closest number to 1000 that is evenly divisible by 13) evenly spread across the temporal range (13 years) for a total of 77 papers per year. Overall, R code was only available for 55 of the 1001 papers examined (5.5%; Figure 1). When shared, code was most often in the Supplemental Information (40%), followed by Github (22%), Figshare (11%), or other repositories (37%). The majority of code (67%) did not include a license. Where a license was included, it was nearly always permissive or copyleft (e.g., CC0, CC‐BY, GPL, and MIT), with only one publication including a proprietary license. Open‐access publications were twice as likely to share code than closed‐access publications (8.5% vs 4.24%, *Χ* ^2^ = 7.2576, *p* = .008599). Publications with open data were 12 times more likely to share code than closed‐data publications (26.5% vs 2.2%, *Χ* ^2^ = 133.36, *p* = 9.999e‐05). Among the set of publications that did not cite R, 6.2% mentioned using R in the text. Of the 33 papers that shared code via potentially citable DOIs or URLs, we were unable to find any citations of the code itself.

### 3.1 Code sharing over time

The proportion of publications sharing code has increased significantly (*p* = .00157) over time (Figure 1, Table 2), with code sharing increasing at an average of 0.6% per year over this period. A Durbin–Watson test indicated no temporal autocorrelation in residuals (DW = 1.7544, *p* = .6475). We note that the years of 2021 and 2022 showed notable shifts toward more frequent sharing (although 2013 showed a similar level of code sharing), but the percentage of code sharing has been consistently below 20% over the past decade, and has remained lower than the percentage of open‐access papers or papers sharing data (Figure 1). Over this same time period, the proportion of publications including data also increased significantly (*p* = 1.48e‐06; Figure 1), while the proportion of open‐access publications did not change significantly (*p* = .926; Figure 1).

**TABLE 2:** Estimated coefficients for models of temporal trends in code sharing, data sharing, and open access over time.

Responses	Parameters	Estimates	Std. error	*z*‐Value	*p*‐Value
Code sharing	Intercept	−3.68612	0.32626	−11.298	<2e‐16***
	Year (relative to 2010)	0.12443	0.03936	3.161	.00157**
Data sharing	Intercept	−2.68284	0.21073	−12.731	<2e‐16***
	Year (relative to 2010)	0.12561	0.02609	4.814	1.48e‐06***
Open access	Intercept	−0.88778	0.13130	−6.761	1.37e‐11***
	Year (relative to 2010)	0.00172	0.01855	0.093	.926

***p < .001, **p < .01.

### 3.2 Impact of code sharing on citations

Our best‐performing model of citation counts (ID 1; Table 1) had a substantially better AIC score than our other candidate models (ΔAIC ≥834.0). Impact factor (*β* = .326398, *p* < 2e‐16), age (*β* = .626261, *p* < 2e‐16), and open access (*β* = .069522, *p* = .000628), all had significantly positive associations with the number of citations, while code availability (*β* = −1.425857, *p* < 2e‐16) and open data (*β* = −.07596, *p* = .025014) both showed significantly negative associations (Table 3). Interactions between age and both code availability (*β* = .562521, *p* < 2e‐16) and data availability (*β* = .212861, *p* < 2e‐16) were positive and significant, while interactions between age and both impact factor (*β* = .001585, *p* = .803079) and open access (*β* = .022175, *p* = .20503) were not significant. Pairwise interactions between the three open‐access criteria were all positive and significant: code availability and data availability (*β* = 1.468237, *p* < 2e‐16), code availability and open access (*β* = 1.454602, *p* < 2e‐16), and open access and data availability (*β* = .174743, *p* = 5.08e‐05). The overall model had a pseudo‐*R* ^2^ of .93.

**TABLE 3:** Estimated coefficients for the selected model predicting number of publication citations (ID 1 in Table 1). Bold entries indicate significance at *α* = .05.

	Estimate	Std. error	*Z*‐value	Pr(>|*z*|)
**(Intercept)**	**2.696757**	**0.011259**	**239.52**	<2e‐16***
**Impact factor**	**0.326398**	**0.006472**	**50.429**	<2e‐16***
**Age**	**0.626261**	**0.010294**	**60.837**	<2e‐16***
**R code shared (yes)**	**−1.425857**	**0.094131**	**−15.148**	<2e‐16***
**Open access (yes)**	**0.069522**	**0.020333**	**3.419**	.000628***
**Data shared (yes)**	**−0.07596**	**0.033893**	**−2.241**	.025014*
Impact factor: Age	0.001585	0.006358	0.249	.803079
**Age: R code shared (yes)**	**0.562521**	**0.037058**	**15.179**	<2e‐16***
Age: Open access (yes)	0.022175	0.017498	1.267	.20503
**Age: Data shared (yes)**	**0.212861**	**0.02383**	**8.932**	<2e‐16***
**R code shared (yes): Data shared (yes)**	**1.468237**	**0.069624**	**21.088**	<2e‐16***
**R code shared (yes): Open access (yes)**	**1.454602**	**0.070556**	**20.616**	<2e‐16***
**Open access (yes): Data available (yes)**	**0.174743**	**0.043125**	**4.052**	5.08e‐05***

*Note*: Statistically significant model terms in bold. ***p < .001, *p < .05.

## 4 DISCUSSION

**FIGURE 2:**
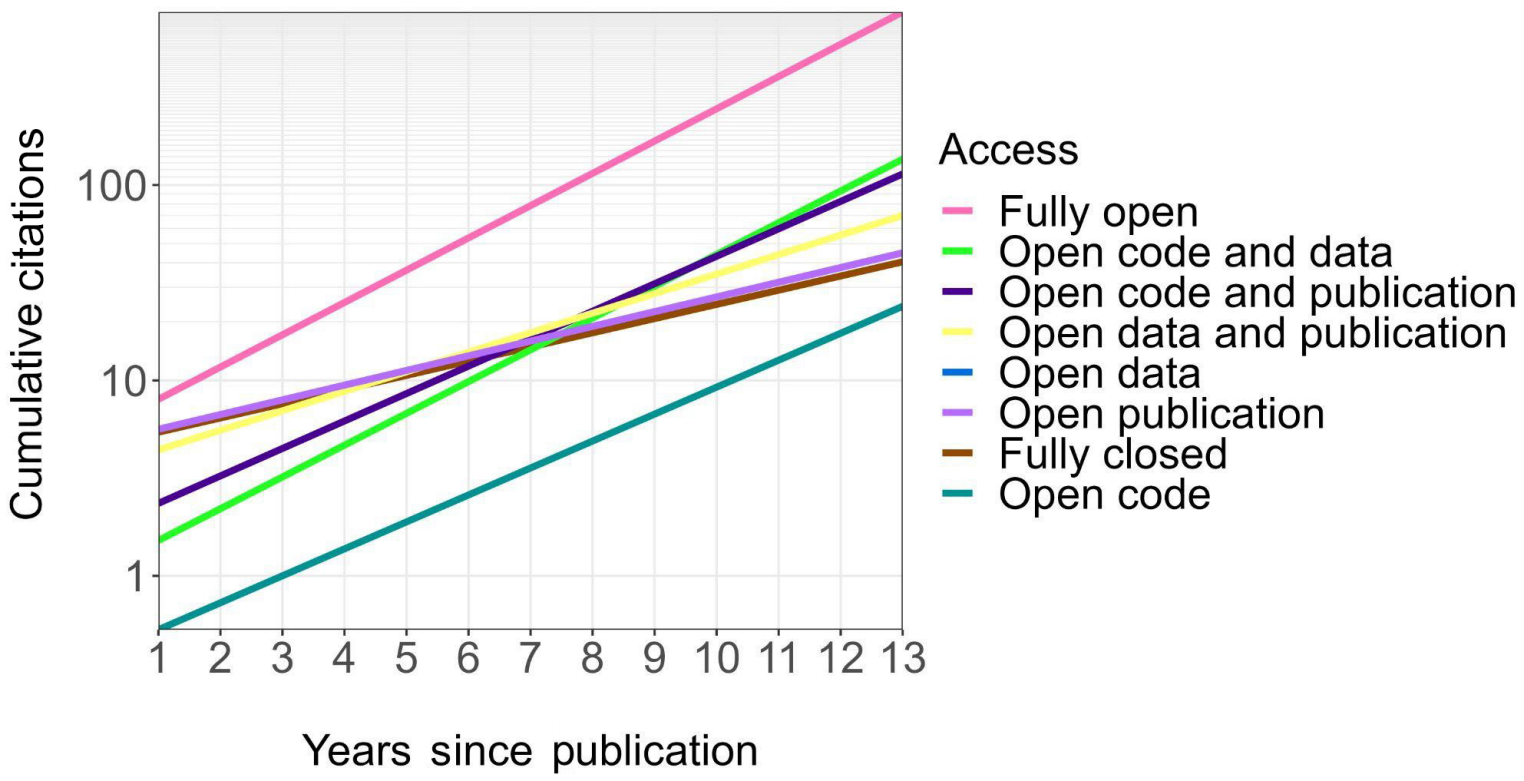
Predicted impacts of code sharing on cumulative citations. Predicted values shown are for the mean impact factor (3.0) across the publications analyzed. Fully open = open code, open data, and open‐access publication; fully closed = closed‐access publication, full data not shared, and no publicly available code. Predictions are based on estimated model coefficients (Table 3). Note that the open‐data line is hidden by the open‐publication line.

We found that the scientific literature in ecology and evolution still falls far short of the code sharing required for adequate reproducibility and transparency, despite an increasing trend in code sharing over the last 12 years. This low rate of code sharing undoubtedly hinders scientific progress and likely has far‐reaching financial consequences, since a lack of reproducibility means that coding must be continuously redone for common analytical tasks (Freedman et al., 2015). Further, our results indicate that the failure to share code may also reduce the academic impact of scientists, as sharing code leads to a higher rate of citation accumulation (i.e., a significant year‐by‐code‐sharing interaction; *p* < 2e‐16; Table 3). The positive effect of code sharing is increased when other open‐science criteria are also met, as evidenced by significant, positive interactions between open access and code sharing (*p* < 2e‐16; Table 3) and between data sharing and code sharing (*p* < 2e‐16; Table 3), with publications meeting all three open science criteria (code sharing, data sharing, and open access) having the highest overall predicted citation rates (“Fully open,” Figure 2). Surprisingly, our model found negative effects of code sharing and data sharing on citation count, despite the positive impacts of code sharing and data sharing on citation rate (i.e., significant interactions between code or data sharing and age). One possible cause of this discrepancy could be that scientists may be less likely to share code and data underlying publications they expect to be impactful if they are planning related studies using the same code or data. Alternatively, this discrepancy may be due to the increase in code availability with time leading to a disproportionate amount of the papers which share code being young, and hence having few citations. We also did not find support for an interaction between publication age and impact factor, suggesting that though impact factor may affect the total number of citations, it does not strongly affect the citation rate. We also did not find evidence of a significant interactive effect of publication age and open access on citation count. This failure of open access to impact citation rate, as well as the lack of change in the percentage of open access publications over time (Figure 1) may be driven by changes to open access status of publications over the years. As journals switch to open access, many papers that were initially published closed access have been converted to open after varying lengths of time, potentially degrading the signal of open access on citation counts.

**FIGURE 3:**
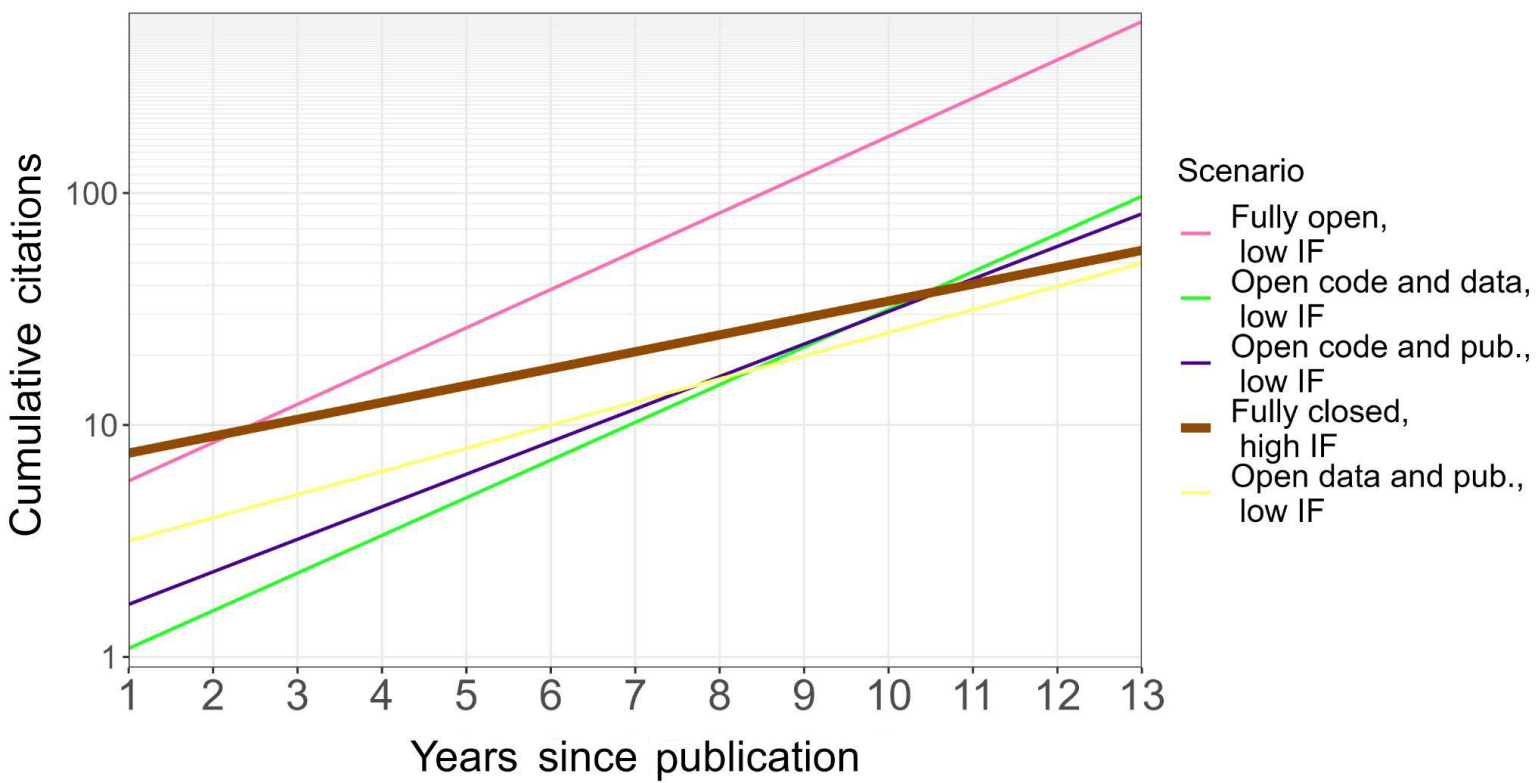
Code sharing can balance out low impact factors. Low impact factor (IF; 1.3) and high impact factor (4.7) are defined using the 0.1 and 0.9 quantiles in our dataset. Fully open = open code and open access publication; fully closed = closed‐access publication and lack of publicly available code. Predictions are based on estimated model coefficients (Table 3). Our model predicts that 3 years after publication, fully open papers published in a low impact journal may have roughly the same number of citations as fully closed papers published in high impact journals (on average). Legend is arranged in descending order of citations at year 13.

Our results suggest that scientists and journals who embrace code sharing may be more impactful, even if the publications are closed‐access. For instance, our model predicts that a scientist who publishes a paper sharing code and data in a journal with an impact factor of 3 will have approximately double the citations after 11 years (64.1 vs 29.0) than if they had published without sharing code and data (Figure 2). However, if this same paper is also published open‐access, our model predicts it will have twice as many citations after only 3 years (17.1 vs 7.6, Figure 2). Further, although papers in high‐impact journals may be expected to receive more citations in general than those in low‐impact journals (although note that the impact factor by age interaction was not significant), our model suggests code sharing may balance this out, particularly when combined with other open‐science factors. By the third year following publication, a fully open publication in a low‐impact (10th percentile, impact factor = 1.3) journal was predicted to have more citations than a fully closed publication in a high‐impact (90th percentile, impact factor = 4.7) journal (12.3 vs 10.6, Figure 3). For publications in low‐impact journals sharing code and meeting only one other open science criterion (open access or data sharing), the fully closed publication in a high‐impact journal is predicted to be surpassed by the 11th year (45.8 and 42.6 vs 40.5; Figure 3).

Our finding of a significant effect of code sharing on citation rate is surprising given that most code was not licensed for reuse. Software licenses play a critical (and often unappreciated) role in code sharing: permissive licenses (e.g., MIT) encourage reuse, while restrictive or proprietary licenses can still allow methodological transparency while limiting or preventing the reuse of published code (Stodden, 2009). Importantly, where code is published without a license, the author retains the copyright (Stodden, 2009). The finding of a significant effect of code sharing on citation rate despite the rarity of permissive software licenses may indicate that the citation benefits for code‐sharing publications are due to either (1) code being reused without proper licensing, or (2) availability of code increasing citations in the absence of code reuse. The latter could happen if code availability increased confidence in the findings of a publication. Given the increasing importance of code in ecology and evolution (Feng et al., 2020), both scientists and funding organizations need to give more thought to software licenses. We note that considering licensing is important both for scientists who wish their code to be freely available and benefit from citations stemming from reuse as well as for scientists who wish to embrace transparency without allowing use of their code.

We note that there are important limitations to our study. The low rates of code sharing and data sharing limited our sample sizes which in turn likely impacted model precision. These low sample sizes may help explain the anomalously high number of publications sharing code in 2013 (Figure 1). Further, these low rates of sharing led to a major imbalance in our main variable of interest, code sharing (55 that shared vs 946 that did not). We also note that while we treated code and data sharing as binary variables, there is a tremendous amount of variation in the amount and quality of data and code that are shared. Where some publications include only summary data or example code, others include well‐documented code and data, and this variation could impact citation count. Some of the variables we examined may change over time, potentially weakening inferences: archived data and code may be lost, publications may become open access, and impact factors change over time. Our search was limited to English language publications, so these trends may not hold for publications in other languages (Konno et al., 2020), though they may be expected to share code even less often (Serwadda et al., 2018). Further, our work focused on papers that cited R, and does not account for the small percentage of papers which note using R but do not cite it. Thus, our results may overestimate the proportion of total publications around the globe sharing code. Finally, our work focused on one programming language, R, which may not be broadly representative.

To create an environment conducive to reproducibility and transparency, we call upon scientists, funding sponsors, publishers, and institutions to champion code sharing and acknowledge its role as a valuable and necessary contribution to the scientific process. Major funding sponsors are beginning to mandate open science approaches (e.g., the EU's Horizon Europe: https://research‐and‐innovation.ec.europa.eu/funding/funding‐opportunities/funding‐programmes‐and‐open‐calls/horizon‐europe_en, and the US Year of open science: https://open.science.gov/). Scientific journals can also play a significant role in this transformation (McNutt, 2014; Mislan et al., 2016; Nosek et al., 2015; “Reality check on reproducibility”, 2016). Minimally, journals facilitate the deposition of code in a stable repository and provide a link to that repository (Mislan et al., 2016; ideally in both human and machine‐readable formats; Peng, 2011). Unfortunately, even when funders and journals mandate data and code sharing, compliance often remains low (Culina et al., 2020). More ambitious solutions might include incorporating links between methods text and the corresponding code, employing dedicated code editors to help improve code style and clarity (similar to Data Editors employed by The American Naturalist), or incorporating computational notebooks (e.g., RMarkdown, Quarto, Jupyter; Peng, 2011). Such measures will enhance transparency in reporting and provide reviewers and readers with the critical information necessary to reproduce and validate the study's findings. The adoption of these principles and practices will serve to promote the integration of open code in the scientific landscape, enhancing the verifiability and impact of our research.

Given the growing list of reasons for code sharing, we encourage scientists to embrace open code and open science more generally. Although maintaining well‐documented code in a version‐controlled public repository (e.g., Github) and public archive with directions for its use (e.g., Zenodo) is ideal for code sharing, other options that require less effort can at least ensure the distribution of code to other researchers interested in using it. Recent advances in artificial intelligence (e.g., ChatGPT) have made documenting scripts easier, thus lowering the cost to authors to share documented code (Merow, Serra‐Diaz, et al., 2023). Finally, we stress that as code sharing increases, our attribution practices must keep pace, both for scientific transparency and to credit the developers (Merow, Boyle, et al., 2023).

## AUTHOR CONTRIBUTIONS

**Brian Maitner:** Conceptualization (lead); data curation (lead); formal analysis (lead); investigation (lead); methodology (lead); project administration (lead); resources (lead); software (lead); supervision (equal); visualization (lead); writing – original draft (lead); writing – review and editing (equal). **Paul Efren Santos Andrade:** Investigation (supporting); writing – original draft (supporting); writing – review and editing (supporting). **Luna Lei:** Formal analysis (supporting); investigation (supporting); methodology (supporting); software (supporting); writing – original draft (supporting); writing – review and editing (supporting). **Jamie Kass:** Conceptualization (supporting); methodology (supporting); writing – original draft (supporting); writing – review and editing (supporting). **Hannah L. Owens:** Conceptualization (supporting); methodology (supporting); writing – original draft (supporting); writing – review and editing (supporting). **George C. G. Barbosa:** Writing – original draft (supporting); writing – review and editing (supporting). **Brad Boyle:** Conceptualization (supporting); methodology (supporting); writing – original draft (supporting); writing – review and editing (supporting). **Matiss Castorena:** Conceptualization (supporting); methodology (supporting); writing – original draft (supporting); writing – review and editing (supporting). **Brian J. Enquist:** Conceptualization (supporting); methodology (supporting); writing – original draft (supporting); writing – review and editing (supporting). **Xiao Feng:** Conceptualization (supporting); methodology (supporting); writing – original draft (supporting); writing – review and editing (supporting). **Daniel S. Park:** Conceptualization (supporting); methodology (supporting); writing – original draft (supporting); writing – review and editing (supporting). **Andrea Paz:** Conceptualization (supporting); methodology (supporting); writing – original draft (supporting); writing – review and editing (supporting). **Gonzalo Pinilla‐Buitrago:** Conceptualization (supporting); methodology (supporting); writing – original draft (supporting); writing – review and editing (supporting). **Cory Merow:** Conceptualization (supporting); methodology (supporting); writing – original draft (supporting); writing – review and editing (supporting). **Adam Wilson:** Conceptualization (supporting); funding acquisition (lead); methodology (supporting); project administration (lead); supervision (lead); writing – original draft (supporting); writing – review and editing (supporting).

## CONFLICT OF INTEREST STATEMENT

The authors declare no competing interests.

### OPEN RESEARCH BADGES

This article has earned Open Data and Open Materials badges. Data and materials are available at https://doi.org/10.5281/zenodo.8201251.

## Data Availability

All code and data underlying this work are available on Github at https://github.com/bmaitner/R_citations and are permanently archived at https://doi.org/10.5281/zenodo.8201251.
